# Advice-seeking and advice-giving in Arabic computer-mediated communication in the medical context

**DOI:** 10.3389/fpsyg.2023.1070310

**Published:** 2023-09-22

**Authors:** Naif Barakah Al-Mutairi, Mohammad Mahzari

**Affiliations:** Department of English, College of Science and Humanities in Al-Kharj, Prince Sattam Bin Abdulaziz University, Al-Kharj, Saudi Arabia

**Keywords:** advice-seeking, advice-giving, CMC, Arabic, patients, doctors

## Abstract

The current study aims to examine the discourse patterns and strategies of advice-seeking and advice-giving through Arabic computer-mediated communication (CMC) in the medical context. The contribution of this study lies in examining and analyzing the discourse patterns of CMC between patients and doctors in Arabic, how these patterns help doctors understand the advice sought and inquiries made by their patients, and how they help patients understand the comments made by their doctors on their inquiries. The data consist of 300 messages categorized into 150 advice-seeking (postings) and 150 advice-response (replies) messages. They were manually collected from a public medical website by copying and pasting them into an Excel spreadsheet for coding and statistical analyses. Two models were used to analyze the speech acts of advice. Morrow’s model was used for advice-seeking, while Hinkel’s taxonomy was used for advice responses, after some modifications were made to both models. The findings revealed that advice-seeking and advice-response messages had three parts: opening, middle, and closing. The study revealed that both patients and doctors used the opening and closing parts occasionally, unlike the middle part, which was used more often. For advice-seeking, describing the medical problem was the most frequent strategy used by patients when they asked for advice, followed by yes/no questions. These types of strategies were used in advice-seeking more frequently than others. Additionally, it was found that asking yes/no questions and describing the medical problem was the most frequent compound strategy used by patients seeking advice. However, for advice responses, the results revealed that giving clarification/information was the most frequent strategy used by doctors, followed by direct advice. When compared with other types, these two types were used frequently in the advice responses. The results also demonstrated that giving clarification/information and direct advice was the most frequent compound strategy used by doctors in advice responses.

## 1. Introduction

In recent decades, with the development of the Internet in terms of computer-mediated communication (CMC) modes, patients do not have any longer to seek advice from doctors in clinics. Instead, they have started using websites for several reasons. For instance, it is easier than going to clinics, which saves time and gives patients an opportunity to ask about anything they may want to know because of increased privacy. No earlier study has attempted to investigate advice-seeking and advice responses in Arabic through CMC in a medical context, despite the importance of this field, which reflects people’s healthcare concerns in terms of communication between patients and doctors on online platforms. Therefore, this study aims to examine the discourse patterns and strategies of advice-seeking and advice-giving in Arabic through CMC in a medical context by adapting Morrow’s (2006) model and Hinkel’s (1997) taxonomy. The strategies of advice-seeking are the methods that the patients use when they ask for advice, whereas the strategies of advice-giving are the means that the doctors employ when they give advice to patients. The compound strategies show the common strategies that are used together for each advice-seeker and advice-giver and their frequency. Morrow’s (2006) model was used for advice-seeking, while Hinkel’s (1997) taxonomy was used for advice responses. Morrow (2006) analyzed the content of messages posted on a website about depression in terms of discourse patterns and features, which were classified into three types: problem messages (e.g., openings and closings, describing the problem, expressing feelings, and types of questions in requesting advice), advice messages (e.g., solidarity and positive regard, direct imperatives to indirect suggestions and hints), and messages of thanks. Regarding the types of questions, Morrow (2006) interpreted the use of yes/no questions when the patient offered one or two answers with some competence in dealing with his/her problem. Therefore, the questions were classified into two types: yes/no questions and WH questions because of the discourse functions and meanings in the medical context in the current study. In contrast, Hinkel (1997) analyzed the responses related to advice-giving and explored three types (e.g., direct, indirect, and hedged advice), and she expanded the analysis to identify the types of hedges in advice (e.g., possibility hedges) (Hinkel, 2005). The models were employed and modified to cover the new discourse patterns and strategies in both advice-seeking and advice-giving in the Arabic online medical context. Earlier studies in Arabic on the discourse realization of speech acts in the medical context focused on the advice that is used in Arabic and English TV health programs (Ahmed and Ahmed, 2019) and what advice-giving strategies the departments of health in Saudi Arabia and Australia provided to the public during the COVID-19 crisis on Twitter (El-Dakhs, 2021) but not on medical websites. Therefore, the current study is distinguished by its focus on advice-seeking by patients and advice-oriented responses by doctors on Arabic medical consulting websites with the aim of answering the following questions:

1. What are the discourse patterns and strategies of advice-seeking in the online medical context?
2. What are discourse patterns and the strategies of responses to advice-seeking in the online medical context?
3. How do the advice seekers and givers construct the message in the online medical context?

## 2. Theoretical background

### 2.1. Speech act theory

The theory of speech acts was developed and originated by Austin; later, Searle (1969) developed the theory further into more speech acts inspired by Wittgenstein’s use theory, Austin’s speech act theory, and Grice’s implicatures theory. Speech act theory is concerned with how the speaker can make his/her communicative goal clear through speech and how the listener can recognize and understand that intention (Austin, 1962; Searle, 1975). Austin (1962) claims that a speech act is a speaking unit that performs specific functions and that a single speech act can be further divided into three other acts: a locutionary act (the main act of speaking), an illocutionary act (when someone says something with a specific purpose with reference to the communicative intention of the speaker’s mind), and a perlocutionary act (refers to the effect of an utterance on the addressee).

Searle (1968) argued that sentences can have different strengths but one literal meaning. For example, according to context, they can have the force of a promise, prediction, threat, or warning, and “one and only one literal meaning” (Searle, 1968, p. 406). Speech acts are not merely spoken words. However, they are verbal actions that occur worldwide. This means that when someone says something, he/she does something using his/her words. Therefore, speech acts imply that the speaker performs an action that changes the current state of affairs (Mey, 2001, p. 96). Fought (2006) indicated that a speech act is an utterance that constitutes a social action. For instance, “give me that” is a directive speech act in which the speaker directs the receiver to perform an act. When utterances contain threats or elements of physical or mental harm, the speech act theory is appropriate to use (Haiman, 1993).

### 2.2. Speech act of advising

The speech act of advice is considered a face-threatening act (FTA) to people who receive the advice. Brown and Levinson (1987) employed the notion of face and clarified that every person has two types of face: positive and negative. A “positive face” refers to the desire to be approved of or appreciated by others, whereas a “negative face” refers to the freedom of action or the desire not to be imposed upon by others. Therefore, advice-givers tend to use various discourse strategies to make the advice seem acceptable by reducing the threat, such as using hedges, indirect advice, positive regard, etc. for politeness purposes. Holmes (1995) defined politeness as “an expression of concern for the feelings of others” (p. 3), and people can express their concern for others verbally and non-verbally. Spiers (1998) pointed out that politeness is used mainly to ease social interaction by providing a ritualistic form of verbal interaction that reduces the severe nature of many speech acts, such as commands, requests, questioning, advice, etc. In other words, the speaker maintains both his/her face and the face of the addressee in social interactions.

From the perspective of speech act theory, Searle (1969) defined the speech act of advising as “telling you what is best for you” (p. 67). He also, in his classification, classified advising within the directive category. Therefore, as a directive speech act, advising means that the recipient is required to perform an action according to the speaker’s desire; however, this action is for the sake of the recipient. The speech act advising implies the speaker’s authority and expertise and the recipient’s need for advice. Therefore, advising is different from other speech acts because it leaves acceptance to the recipient (Bouwmeester, 2010).

DeCapua and Dunham (1993) define advice as “opinions or counsel given by people who perceive themselves as knowledgeable, and/or who the advice seeker may think are credible, trustworthy, and reliable” (p. 519). Moreover, Tsui (1994) defines advice as “a directive which advocates a course of action for the benefit of the addressee, and in which the consequence of compliance is desirable” (p. 122). Regarding the theoretical concept of advice in the medical context, Van Poppel (2019) specified four felicity conditions for advising in the context of health:

1. Essential condition: Advising concerns an attempt by the advisor to make the receiver perform a beneficial act to treat or prevent a problem that might affect the latter’s health.
2. Propositional content condition: The advisor predicates a future act that is beneficial to the receiver’s health.
3. Preparatory conditions:
  - The advisor has some reason to believe that the act will be useful for the receiver’s health by preventing, treating, or detecting a health problem.
  - The advisor believes that the receiver is willing to perform the action.
  - The advisor believes that the receiver is able to perform the action.
  - The advisor is a health authority with knowledge and experience of the action and its effects.
  - It is not obvious to both the advisor and receiver that the receiver will perform the action during the normal course of events.
  - The advisor believes that the receiver has not yet done or is not yet performing the action.
4. Sincerity conditions:
  - The advisor wants the receiver to do the action.
  - The advisor believes that the action benefits the receiver’s health.

Mićic (2013) indicated that the medical language is a register used by doctors; hence, it seems like an odd language outside the context of medicine. According to Bloom (1982), “medicine” can be defined as the science of preventing, treating, and diagnosing diseases. It also refers to the drugs used to treat any disease or injury. There are some characteristics of medical language, such as the extensive use of words that are largely related to the subject matter. This means that each word, phrase, or sentence uttered in the medical context is special and refers to medical jargon. In addition, medical language is characterized by the use of passivization and an impersonal style. Therefore, specialists use third-person rather than first-person pronouns (Mey, 2009). However, it is expected that a simple language used by doctors and patients will be understood.

## 3. Literature review

### 3.1. Advice in computer-mediated communication

Several studies have been conducted to investigate the speech act of giving health advice in different contexts, including radio advice programs (DeCapua and Dunham, 1993), TV health programs (Ahmed and Ahmed, 2019), Twitter (El-Dakhs, 2021), and online forums (Morrow, 2006; Kouper, 2010; Locher, 2013; Pung, 2017; Bates, 2021). For example, in the context of radio advice programs, DeCapua and Dunham (1993) examined the patterns in American English used by speakers (adults) when requesting and giving advice. The researchers collected data through calls from two different radio advice programs. The results showed that explanation, elaboration, and narration were the main strategies used by speakers when requesting and giving advice. These strategies were used by people to successfully interact with each other. It was observed that before advice-givers gave advice, callers gave a long clarification for the problem. Among these strategies, this study pointed out that narration was used more by both advice-seekers and advice-givers. The results also indicated that requests for advice by advice-seekers were implicit, sometimes vague, or included no requests at all. This is because the advice-seeker expects the request to be evident from the description of the problem. On the TV health programs, Ahmed and Ahmed (2019) examined the advice given in Arabic (Green Apple) and English (doctors) programs. The results showed that all pieces of advice in the two programs were used in polite expressions, which reflects the strong relationship between advising speech acts and politeness. The results also indicated a difference between the two programs regarding the types of politeness employed. It was found that in the English program, positive politeness strategies were used in all texts. However, in the Arabic program, negative politeness strategies were mostly used. In addition, the data clarified that in the English program, imperative sentences were used more than declarative ones, whereas in Arabic, the data showed more use of declarative sentences in comparison with imperative ones.

During the COVID-19 crisis on Twitter, El-Dakhs (2021) analyzed what advice-giving strategies the departments of health in Saudi Arabia and Australia provided to the public. The results showed that direct advice strategies were the most preferred among both Saudis and Australians. The use of direct advice strategies is preferred in the health domain. In terms of differences, the results indicate that Saudis preferred to use more direct advice strategies and external modifiers, whereas Australians used more conventional indirect strategies and internal modifiers. It also clarified that the Saudi health department, in terms of modifiers, used more religious expressions, whereas the Australian health department used some downgraders to try to sound reader-friendly and create engaging discourse.

Among peer advice interactions in an online community at LiveJournal.com on the topic of motherhood, Kouper (2010) studied how people take part in soliciting and giving advice, what strategies are used, and how they deal with the potential face threats in advice exchanges. The results showed that requests for information or opinions were the most common type of advice solicitation, followed by problem disclosures. It was also pointed out that requests for advice and problem disclosures were identified as systematically occurring in the data: “orientation, justification, and appreciation.” The use of these elements can vary frequently in terms of the frequency and positioning in the message. Therefore, messages that solicit advice vary in length, content, and structure. Such messages are often written in a narrative manner. It is often found that one message contains several questions or requests for advice. In addition, the results indicate that people in this community used all four strategies (hedged advice, direct advice, indirect advice, and descriptions of personal experiences) in advice-giving. The most common forms of advice were direct advice and sharing of personal experiences. However, in cases where there is greater sensitivity, people share personal experiences more often than hedged or indirect advice.

In an online forum among Malaysian women, Pung (2017) examined advice-seeking and advice-giving strategies and the influence of their culture. The results showed that the most frequently used strategies for advice-seeking were question-asking, followed by problem description, whereas an explicit request for advice was the least preferred strategy. However, for advice-giving strategies, the most preferred were giving or offering advice directly, followed by giving indirect advice, describing one’s own experiences, and providing general information. The findings also showed that the examination of many aspects of the interactions among women revealed participants’ cultural influence at the level of message content as well as the discourse.

Another study on an online forum, Bates (2021) investigated the mitigation strategies used when requesting and providing advice for recovery for people with eating disorders. The results showed that participants in the forum used different mitigation patterns for advice-seeking and advice-giving in the forum. For advice-seekers, the results indicated that mitigation devices and discursive moves were used to minimize the illocutionary force of a request and cope with the stress of the disorder. It was noticed that instead of directive acts, hedges were the most common mitigating devices in the data and were used by advice-seekers to soften the force of the request. On the other hand, for advice-givers, the findings revealed that affective appraisals, personal experience, empathy displays, and concessive patterns were the main discursive moves used by advice-givers to soften the message, express empathy, and reduce the advisee’s psychological vulnerability. Overall, it was observed that mitigation is an important way to deal with multiple stressors and multidimensional vulnerabilities in discourse.

Another communication context that receives attention in advice studies is the health domain. For example, Locher (2013) focused on computer-mediated advice regarding health issues and collected data from question-and-response letters in an online archive. An analysis of the data showed that the texts were segmented into “discursive moves.” The letters contained several parts, such as the problem, general information, explanation of points made, and farewells. While the analysis of the responses pointed out that there was both direct and indirect syntactic advice-giving, advice was included within the entire composition of the letters where the advice-seekers could make their own decisions by being given options. In addition, Morrow (2006) studied the discourse features of messages (postings and replies) posted on an Internet discussion forum on the topic of depression. The researcher classified these messages into three categories: problem messages (describing a problem), advice messages (responding to the problem messages), and thanks messages (written in response to the advice messages). The results showed that the discussion begins with “a problem message,” i.e., a presentation of the symptom by using metaphorical language to describe the medical problem, types of questions used to request advice, and the lack of direct requests for advice. This is usually followed by one or more advice messages in response. It was observed that sometimes there were many replies to the advice messages, either from the writer of the initial problem message as a “thanks message” or from others as “comments” on the advice messages. The results also indicated that the problem, advice, and thanks messages had similarities in terms of style but differed in terms of their discourse characteristics. In terms of the style, the researcher found that both the problem messages and the advice messages were written in an informal style, including the use of, for example, “u” for “you.” The author discussed three types of messages.

For the first type, Morrow (2006) revealed that problem messages were different in terms of structure and content. Some messages have three parts: an opening, a description of the problem, and a closing. However, not all of these parts could be found in all problem messages. The consistent part was a description of the problem. In addition, the findings indicated that there were relatively few direct requests for advice in “problem messages.” In addition, yes/no questions and WH questions were employed as a request for advice, and Morrow (2006) interpreted the use of yes/no questions when it was employed by patients with some competence in dealing with their problems. A similar use of yes/no questions was observed in Heritage and Sefi’s (1992) study on visiting nurses. They found that direct requests for advice were relatively few, and requests for advice were often in the form of yes/no questions that required confirmation. Heritage and Sefi (1992) explained the reason for using yes/no questions as a result of a desire to appear competent to handle one’s own affairs.

For the second type, advice messages, the results clarified that to make the advice less threatening to those who received it, advice-givers used different strategies to avoid that threat, such as, for example, showing empathy. It was observed that the advice-givers used an expression of empathy (as an opener) in most of the advice messages, saying, for example, “I just wanted to say that I am in the same boat as you.” Other expressions such as “good luck” or “take care” were used more in the closings to communicate a positive regard, and some expressions such as “feel free to talk to me” were used as offers. The results also showed that the majority of the advice-givers mentioned their own experiences before offering advice to advice-seekers. However, sometimes, advice-givers referred to someone else’s experience. In addition, sometimes when advice was not given, the advice-givers felt forced to explain the reason.

For the third type, thanks messages, the results showed that most expressed appreciation for the advice, and those messages were initiated with an expression of thanks in the opening sentence. In some cases, the writers admitted that they had done what was recommended. However, this was done without admitting that their actions aligned with the advice that they had received. Some thanks massages tended to be shorter. Overall, this study suggested that contextual factors affected the use of discourse patterns in terms of what the message writers said, what they avoided, and how they gave and received advice. In addition, Hinkel (1997) analyzed the responses to advice-giving and explored three types of advice-giving: direct, indirect, and hedged. Hinkel (1997) categorized advice that included hedges as hedged advice. In addition, Hinkel (2005) expanded the analysis to identify the types of hedges and relied on the system outlined by Brown and Levinson (1987), Hübler (1983), and Quirk et al. (1985). According to Hinkel (1997), hedges are defined as linguistic mitigating devices, e.g., particles, “tentativizers” (Brown and Levinson, 1987, p. 153), weakeners, and minimizers, employed to redress a potential FTA and reduce the speaker’s responsibility, for his or her cooperativeness, “informativeness, truthfulness, relevance, and clarity which on many occasions need to be softened for the reasons of face” (Brown and Levinson, 1987, p. 146). In addition, Hinkel (2005) analyzed and discussed the types of hedges in English, such as epistemic hedges, lexical hedges, possibility hedges, downtoners, assertive pronouns, and adverbs of frequency, which are affected by context.

Our review of previous studies shows that, to date, no study has attempted to investigate seeking and responding to advice in Arabic in the medical context through CMC, such as the use of a medical website. The current study aimed to fill this gap by investigating the discourse patterns of advice-seeking and advice responses to identify the strategies and structure in Arabic through CMC in the medical context.

## 4. Methodology

### 4.1. Data collection

The data for this study were collected in April 2022 from advice-seeking and advice-response messages on the Altibbi website (https://altibbi.com) about various symptoms in various health contexts, such as surgery, psychiatry, medicine, and ophthalmology. This website is one of the most important medical websites and is presented by a group of medical professionals who answer various health-related questions in Arabic. It is considered the first website in the Arab world to provide online medical consultation and certified information. It is set up as a site where patients with various medical problems can ask doctors about them freely and anonymously. At the time of data collection, the Altibbi website had more than 20 million users, more than 900,000 types of medical information, more than 137,343 doctors in service, more than 3,000,000 medical questions, and 4,350,504 medical consultations. There are more websites, but this one was chosen for the study of advice-seeking and advice responses for several reasons. First, it is certified by the ISO and is licensed. Second, it received the World Summit Award for the best online medical content for Arab readers. Therefore, this website provides a good context for studying advice-seeking and advice responses in the medical context.

The messages analyzed were posted from April 3–5, 2022. On this website, there were two filters. First, when choosing the type of question, we had the option to choose the most recent questions or the most frequently visited ones, but we opted for the former. The second filter was then classified. We did not determine any specific classification based on symptoms. However, we did a general search for all types of symptoms to obtain general data in the field. To obtain reliable results, we collected the data naturally and in the order the responses appeared on the website, without overlooking any questions or choosing any specific field. In addition, although there were some misspelled words in some sentences, no modifications were made. This process was performed manually by copying and pasting the data into an Excel file because of the ease of coding and extracting frequencies. The data consisted of 300 messages, including 150 advice-seeking messages and 150 advice-response messages. The advice-seeking messages were posted by 59 male and 91 female patients between the ages of 15 and 52. Their ages were divided into three groups to categorize the range of ages, and it was then found that the first group included 112 patients between the ages of 15 and 28; the second group included 11 people between the ages of 29 and 39; and the third group included 27 people between the ages of 40 and 52. In contrast, among the advice-responders, there were 123 male doctors, and 27 were female. All the personal information of doctors and patients was omitted in our research to protect their privacy, and we focused only on the language used in advice-seeking and advice responses. However, we only mentioned general types of information, such as age, gender, and the language of advice-seeking and advice responses. Although the website is public, and it requires no consent to access the data, the Institutional Review Board (IRB) at our university approved the study based on the procedures for collecting data and protecting the privacy of users of the website.

The advice-seeking messages were sent by patients to doctors asking for help with their problems, while the advice-response messages were sent by doctors in response to the advice-seeking messages. Those messages were written in Arabic except the use of some medical terms, whether by the patients or doctors, which were in English, such as the words “cloraxene,” “trichotillomania,” and so on. For advice-seekers, we included a header with age and gender identified but not names or photos. In addition, we included a classification that indicates what field the problem pertains to. In addition, there were topics and advice-seeking icons. However, we found that the topic-and advice-seeking icons usually had the same expressions used by patients or completed each other as one explanation. Therefore, in the analysis, we considered the topic and advice-seeking icons to be comments. On the other hand, for the advice-response messages, there were gender-and advice-response icons. Although the advice-seeker often received only one reply from a doctor, four advice-seekers received two replies from different doctors. Therefore, we relied on the first replies and neglected the second.

### 4.2. Procedure

Two models were used in this study. First, Morrow’s (2006) model of code classification was used to analyze the data obtained in terms of the strategies of the speech act of advice-seeking. However, we found that some strategies were not available in our data, such as the advisory giver’s stance. In addition, we found that new expressions, such as “suffering from” or religious expressions, required new codes for strategies. These differences were due to the nature of the data as well as the Arabic context. Therefore, we used Morrow’s (2006) model, with some modifications, which was appropriate for Arabic data. In contrast, to analyze advice responses, Hinkel’s (1997) taxonomy of advice was used, which is divided into three classifications: direct advice, hedge advice, and indirect advice. However, the third classification (indirect advice) was not available because of the nature of the data and the medical context, as patients come to the site specifically to ask for help. Therefore, doctors, in giving advice, should use either direct advice or hedge advice.

Several steps were taken to analyze the postings of the speech act of advice-seeking and advice-response messages in terms of their strategies and construction. The data were collected by copying and pasting them into an Excel file to be coded based on Morrow’s (2006) and Hinkel’s (1997) work.

Reliability was established by the researchers. The first author analyzed the first 50 advice-seeking posts and the first 50 replies given independently, and they were revised by the second researcher to work out and agree upon a set of criteria for identifying the strategies employed in the messages. Discrepancies were resolved through discussion by adding new codes owing to the nature of the data. The same procedure was performed with the rest of the posts and replies in terms of analyzing, revising, and resolving discrepancies through discussion.

## 5. Results

The results were divided into two sections: qualitative and quantitative. In the first section, examples of interactions between the patient and doctor are discussed in depth. In the second section, the focus will be on the types of strategies as well as the types of compound strategies employed in advice-seeking and advice-giving by patients and doctors.

### 5.1. Qualitative analysis

The following are some examples of the interactions that took place between patients and doctors in Arabic on the medical website. Two of these interactions are taken from the same situation: one example is a posting by the patient, and the other is a reply by the doctor. The Arabic text is followed by a transliteration (see Appendix A for Arabic symbols) and translation.

In Example 1, the patient began his posting by describing the medical problem: “My cumulative blood sugar is 5.9.” This was followed by using a yes/no question, such as “Am I diabetic?” There was no use of an opening or closing. In the response, the doctor started the reply by using “My dear brother” as an opening, followed by giving clarification/information as in “you are in the pre-diabetes stage, and you do not need treatment.” This was also followed by giving direct advice: “you must commit to improving your diet and exercise,” which was used to conclude the reply. Doctors employed various imperatives either with or without using “should” (see Table 1 for more examples).

**EXAMPLE 1:** 

Male patient:	السكر التراكمي عندي 5.9 هل كدا انا مريض سكر
Transliteration:	al-sukkar al-trakumi ^c^indi 5.9 haL kitha ʔana mari:d sukkar
Translation	My cumulative blood sugar is 5.9. Am I diabetic?
Doctor’s response:	اخي الكريم انت في مرحله ما قبل السكر ولا تحتاج الى علاج ولكن يجب عليك الاتزام بالحميه الغذائيه وممارسه الرياضه على الاقل رياضه المشي ١٥٠ دقيقه اسبوعيا واعادة التحليل بعد ٣ اشهر
Transliteration:	ʔakhi al-kari:m ʔant fi: marhalah ma qabl al-sukkar wala tahtaj ʔila ^c^ilaj walakin yajib ^c^alayk al-ʔiltizam bi al-himyah al-ghithaʔiyyah wa mumarasat al-riya:dah ^c^ala al-ʔaqal riyadah al-mashi 150 daqi:qah ʔusbu:^c^iyyan wa ʔi^c^a:dat al-tahli:l ba^c^d 3 ashhur
Translation:	My dear brother, you are in the pre-diabetes stage, and you do not need treatment. However, you must commit to improving your diet and exercise, walking at least 150 min per week and undergoing re-analysis after 3 months.

**Table 1:** Examples of direct advice using imperatives by doctors.

	Arabic examples of direct advice by doctors	Translation
1	برجاء عمل ايكو على القلب	Please do an echocardiogram on the heart.
2	يجب تناول الملينات	You should take laxatives.
3	برجاء اتباع نصائح الدكتور	Please follow the advice of the doctor.
4	مارس الرياضة والتغذية المتوازنة	Practice exercise and balanced nutrition.
5	يجب عليك الالتزام بالحميه الغذائيه	You must stick to the diet.
6	برجاء الرجوع لطبيب الأنف	Please refer to the nose doctor (the otorhinolaryngologist).
7	تحتاج الى استشارة طبيب	You need to consult a doctor.
8	يلزم الإستمرار في العلاج	Treatment must be continued.
9	يجب عمل موجات فوق صوتيه	You should do an ultrasound.
10	اوقفي القرفة فوراً	Stop ingesting cinnamon immediately.
11	تحتاج لمراجعة طبيب الجراحة للفحص	You need to see a surgeon for examination.
12	عليك مراجعة طبيبك	You should see your doctor.
13	يجب تجنب الوقوف لفترات طويله	You should avoid standing for a long time.
14	الرجاء عمل عينه من مكان الغده	Please provide a sample from the gland.
15	يستحسن ان لا يكون مره اخرى	It is better not to be in this situation again.
16	الافضل اجراء فحص عند طبيب عيون	It is best to get an examination by an ophthalmologist.
17	اعتبريها دوره	Consider it a menstrual cycle.
18	!!انتظر الدورة الشهرية التي تنفي الحمل	Wait for the menstrual cycle, which will indicate lack of pregnancy.
19	مارس الرياضة والتغذية المتوازنة وتناول العسل والمكسرات، واستعد للجولة القادمة	Practice sports and balanced nutrition. Eat honey and nuts and get ready for the next round.
20	اعملي التحليل	Do the medical test.
21	لابد من مراجعه طبيب لتقييم الحاله	You must see a doctor to evaluate the condition.
22	يفضل عمل أشعه على الصدر	It is preferred to do a chest X-ray.
23	اسألي في الصدلية	Ask at the pharmacy.
24	لا يجب أن تقلقي فقط خذي مسكنات ألم	Do not worry, just take pain relievers.
25	يفضل أخذها مساءا قبل النوم	It is preferred to take it in the evening before sleep.
26	راجع اختصاصي جراحة	See a surgeon.
27	حافظ على ذلك	Keep it up.
28	لازم يكون تحت اشراف طبي	It must be under medical supervision.
29	حافظ على النظافة	Keep it clean.
30	تابع مع طبيب الجراحة	Follow up with the surgeon.
31	انتظري ٣ أيام	Wait three days.

In Example 2, the patient initiated her posting by requesting direct advice, i.e., “Tell me” and followed it by using a religious expression to thank the doctor in advance for the advice that will be given later, i.e., “may Allah reward you.” The patient followed her thanks by asking a question using a WH question, i.e., “What is the reason for tilting and moving one’s neck involuntarily towards the right or left side and the inability to move it forward when experiencing anxiety and tension?” The doctor began the reply by giving clarification/information, suggesting that a “Clinical examination is important,” followed by giving a direct advice, i.e., “You have to see a neurologist.” Patients used various imperatives when they asked for advice (see Table 2 for more examples).

**EXAMPLE 2:** 

Female patient:	افيدوني جزاكم الله خيرا ما سبب ميلان و تحرك الرقبه لا إراديا نحو الجهة اليمنى أو اليسرى و عدم المقدره على تحريكها للأمام أثناء القلق و التوتر؟
Transliteration:	ʔafi:du:ni jazakum allah khayran ma sabab mayala:n wa taharruk al-raqabah la ʔiradiyyan nahw al-jihah al-yumna ʔaw al-yusra wa ^c^adam al-maqdirah ^c^ala tahri:kiha lilʔamam athnaʔ al-qalaq wa al-tawattur?
Translation:	Tell me, may Allah reward you. What is the reason for tilting and moving one’s neck involuntarily towards the right or left side and the inability to move it forward when experiencing anxiety and tension?
Doctor’s response:	الفحص السريري مهم عليك مراجعة طبيب أعصاب للفحص السريري لإجراء اللازم من تصوير وتحليل للدم وغيره او لتوجيهك للتخصص المعني
Transliteration:	al-fahs al-sari:ri muhim ^c^alayk muraja^c^at tabi:b ʔa^c^sa:b li al-lfahs al-sari:ri li ʔijraʔ al-lazim min taswi:r wa tahli:l li addam wa ghayrih aw li tawji:hik li attakhassus al-ma^c^ni.
Translation:	Clinical examination is important. You have to see a neurologist for clinical examination to undergo the necessary imaging, blood analysis, etc. or get a referral to the relevant specialist.

**Table 2:** Examples of direct ways of asking for advice by patients.

	Arabic examples of direct ways of asking for advice	Translation
1	ارجو نصيحتي في الامر	Please advise me on the matter.
2	ارجو ان تدلوني عليه جزاكم الله كل خير	Please guide me to it and may Allah reward you with all the best.
3	افيدوني جزاكم الله خيرا	Please advise me and may Allah reward you.
4	ارجوكم من فضلكم اجيبوني	Please answer me.
5	forxiga and komb6 ارجو بيان رديل دواء ال	Please explain the medicine of forxiga and komb6.
6	ارجو من حضراتكم تفاصيل الاشعه	I am asking you for the details of the x-ray.
7	عاوز اعرف علاج اللي ف الصورة	I want to know the medicine for the one in the picture.
8	اريد ان افقد بعض من وزني	I want to lose some weight
9	اريد الابتعاد عن التدخين	I want to quit smoking.
10	اريد دوا لتنزيلها كي اصوم ولا اخسر الشهر كامل	I want medicine to reduce it so that I can fast and not lose the whole month (the holy month of fasting, Ramadan).
11	دلوني اش اسوي	Guide me regarding what to do.
12	ارجو الاجابه	Please answer.
13	أفتونا مأجورين	Answer us and may Allah reward you.
14	اجيبوني جزاكم الله خيرا	Answer me and may Allah reward you.
15	اريد بعض النصائح والارشادات وجزاكم الله خيرا	I want some advice and guidance; may Allah reward you.
16	ارجو نصحي بعلاج مجرب للجرثومة	Please advise me with a proven treatment for bacterium.
17	أريد أشياء طبيعيه لا أريد ادوية و شكرا	I want natural things. I do not want drugs, thank you.

In Example 3, the patient began the posting by describing the medical problem: “My eyes were exposed to the mobile flashlight for a long time, more than a quarter of an hour… not the first time.” This was followed by asking yes/no questions, such as “Am I in danger?” The patient concluded her posting by expressing feelings about herself, i.e., “I feel very worried,” which was intensified by using the adverb “very.” The doctor replied to the patient’s question: “Am I in danger?” with a yes/no answer when the doctor said “There is no risk.” At the same time, the doctor’s response can be considered a positive one. In addition, the doctor followed his response “There is no risk” by giving a direct advice: “it is better not to do it again,” in which the doctor advises the patient not to be exposed to the mobile flashlight again. In other examples, the doctors used hedges instead of using only imperatives in their advice, which are examples of hedge advice, as in, for instance, “it is possible to take…,” “you can take…,” “perhaps, you can take…,” etc. (see Table 3 for more examples).

**EXAMPLE 3:** 

Female patient:	تعرضت عيناي لكشاف الجوال لمدة طويلة اكثر من ربع ساعة...وليست اول مرة هل انا في خطر؟...علما باني اجريت عملية تصحيح ابصار من سنة...اشعر …بالقلق الشديد
Transliteration:	ta^c^arradat ^c^aynay li kashshaf al-jawwal li muddah tawi:lah ʔakthar min rub^c^ sa^c^ah…wa laysat ʔawwal marrah…hal ʔana fi khatar…^c^ilman biʔanni ajrayt ^c^amaliyyah tashi:h ʔibsar min sanah…ʔash^c^ur bi al-qalaq al-shshadi:d.
Translation:	My eyes were exposed to the mobile flashlight for a long time, more than a quarter of an hour… not the first time… Am I in danger? … Noting that I underwent a vision correction operation a year ago… I feel very worried.
Doctor’s response:	لا خطر يستحسن ان لا يكون مره أخرى
Transliteration:	la khatar yustahsan ʔan la yaku:n marrah ʔukhra.
Translation:	There is no risk, but it is better not to do it again.

**Table 3:** Examples of hedge advice by doctors.

	Arabic Examples of Hedge Advice by Doctors	Translation
1	Movicol sachets بالإمكان استخدام	It is possible to use Movicol sachets.
2	بامكانك مراجعة الطبيب للنظر في هذا	You can see a doctor to look into this.
3	بعد تمام الالتئام يمكنك اكل ما تشاء	After complete healing, you can eat whatever you want.
4	تقدري تاخذيهم عادي بعد الدورة اذا استمرت معك الاعراض	Normally, you can take them after your period if symptoms persist.
5	يمكن اخذ الشريط الثانى مباشره بعد الاول بدون فتره راحه	You can take the second tablet immediately after the first one without a break.
6	ممكن تتبع فترة التبويض بالعديد من الوسائل	It is possible to track the ovulation period in many ways.
7	ليس ضروري عمل شيء، ربما الماء البارد والابتعاد عن العادة	It is not necessary to do anything, but perhaps you could drink cold water and get away from the habit.
8	ممكن تناول منظمات الجهاز الهضمي	It is possible to take digestive regulators.
9	ربما نحتاج إلى علاج نفسي مكثف	Perhaps we need intensive psychotherapy.
10	ليس له تاثير على مريض الصرع ويمكن استخدامه بامان مع دواء ديباكين ٥٠٠ملجم	It has no effect on epileptic patients and can be used safely with Depakine 500 mg.
11	يمكنك استعمال مخدر موضعي عند ظهور الحكة	You can use a local anesthetic when itching happens.
12	إذا لم تتحسن يمكنك مراجعة طبيبك	If you do not get better, you can see your doctor.
13	يمكن استخدامه بامان مع دواء ديباكين ٥٠٠ملجم	It can be used safely with Depakine 500 mg.
14	يمكن استخدام مقياس حرارة رقمي لتتبع التغيرات الصغيرة في درجة الحرارة	You can use a digital thermometer to track small changes in temperature.
15	يمكنك المتابعة مع معالج نفسي للإستماع لك ومساعدتك	You can follow up with a therapist who will listen and help you.
16	يمكن الاستغناء عن المتحرك بالتثبيت الثابت	You can replace the mobile one (mobile dental braces) with a permanent one.

In Example 4, the advice seeker initiated her posting by giving personal information about her patient, the baby, and mentioned that “my baby is 6 months old, followed by a description of the medical problem but not a general one. However, the mother of the patient used a strategy of “expressing suffering” when she said that “He is suffering from a severe cough with the presence of phlegm” to describe the problem in a very precise way. In addition, the mother of the patient used a WH question, “What is the best medicine for his condition?” The doctor replied to the question directly by giving clarification/ information without using any other strategies, responding with “Bronchicum,” which is a natural cough remedy.

**EXAMPLE 4:** 

Female patient:	طفلي عمره ستة اشهر يعاني من سعال حاد مع وجود بلغم ماهو افضل دواء لحالته
Transliteration:	tifli ^c^umruh sittat ʔashhur yu^c^ani min su^c^a:l ha:d ma^c^ wuju:d balgham ma huwa ʔafdal dawaʔ li halatih.
Translation:	My baby is 6 months old. He is suffering from a severe cough with the presence of phlegm. What is the best medicine for his condition?
Doctor’s response:	Bronchicum
Translation/description:	(It is a natural cough remedy.)

In Example 5, the patient initiated the posting with a greeting, i.e., “Peace be upon you,” followed by a yes/no question: “are my test results normal?” The patient closed the posting with an expression of thanks and wished the doctor well during the holy month of Ramadan. The two strategies employed in the closing are considered positive politeness strategies.

**EXAMPLE 5:** 

Female patient:	السلام عليكم اريد ان اسأل هل نتائج تحاليلي طبيعية ؟؟ و شكرا رمضان كريم
Transliteration:	assalamu ^c^alaykum ʔuri:d ʔan ʔasʔal hal nataʔij tahali:li tabi: ^c^yyah?? wa shukran ramadan kari:m.
Translation:	Peace be upon you. I want to ask, are my test results normal? Thank you, happy Ramadan.
Doctor’s response:	وعليكم السلام ورحمة الله وبركاته رمضان كريم على الجميع عفوا هل تشكون من خفقان ؟ الشعور بالحراره حتى في الاجواء البارده؟ تعرق باطن الكف؟
Transliteration:	wa ^c^alaykum assalam wa rahmatu allah wa barakatuh ramadan kari:m ^c^ala aljami:^c c^afwan hal tashku:n min khafaqan? al-shu^c^u:r bi al-hararah hatta fi al-ajjwaʔ al-baridah? ta^c^arruq batin al-kaf.
Translation:	Peace be upon you and Allah’s mercy and blessings be upon you; happy Ramadan to all. Sorry, are you complaining of palpitations? Feeling hot even in cold weather? Palms sweating?

The doctor replied to the greeting directly: “Peace be upon you and Allah’s mercy and blessings be upon you; happy Ramadan to all” as a response. The doctor followed the greeting response strategy of asking for more clarification by using questions such as “Sorry, are you complaining of palpitations? Feeling hot even in cold weather? Palms sweating?” The doctor employed more than one question to clarify the condition before giving the advice; however, these questions did not receive a response from the patient because of the nature of the website, which provides only a space for the posting and reply, but not additional responses.

The previous strategies employed by patients and doctors in advice-seeking and advice-giving are summarized in the following section to identify the most common strategies used in the online medical context in Arabic. Different strategies are employed by patients and doctors; however, they refer to advice-seeking and advice-giving in this context.

### 5.2. Quantitative analysis

The second section also contains two subsections. The first focuses on the types of strategies as well as the types of compound strategies employed in advice-seeking by patients and how they construct their postings. In the second section, the types of strategies and compound strategies used by doctors in advice responses are explained, and how they construct their replies to the patients’ postings.

#### 5.2.1. Types of strategies employed by patients in advice-seeking

Table 4 shows the types of strategies employed in advice-seeking. The construction of advice-seeking has three main parts: opening, middle, and closing. It was also noticed that opening and closing were used occasionally. As can be seen, the opening includes a greeting, which was used 19 times (5%), as well as the use of an address term, which was used six times (2%). Also, it is obvious that a greeting, such as *asslamu alaykum* (peace be upon you), and address terms, such as *duktu:r* (doctor), were used more commonly by women than men.

**Table 4:** Types of strategies used by patients in advice-seeking.

Types of strategies in advice-seeking	Males	%	Females	%	Grand total	%
Opening						
Greeting	7	4%	12	5%	19	5%
Using address term	2	1%	4	2%	6	2%
Middle						
Describing the medical problem	56	35%	86	36%	142	36%
Asking questions using a yes/no question	25	16%	59	24%	84	21%
Asking questions using a WH question	18	11%	27	11%	45	11%
Expressing suffering	16	10%	16	7%	32	8%
Expressing feelings	15	9%	13	5%	28	7%
Requesting using direct advice	8	5%	11	5%	19	5%
Asking the doctor to look at medical reports/x-ray	8	5%	5	2%	13	3%
Closing						
Thanks	2	1%	4	2%	6	2%
Use of prayers	2	1%	4	2%	6	2%
Grand total	159	100%	241	100%	400	100%

In addition, the results show that in the closing, both expressions of thanks, such as *shukran* (thank you) and prayers, such as *jazak allah khayr* (may Allah reward you well), were used with the same frequency, and were used six times (2%) in terms of the total. They were used more frequently by female than male patients. In this case, prayers are considered a kind of wish in Islamic culture.

In the main part of advice-seeking, it can be seen that describing the medical problem was the most frequent strategy, being used 142 times (36%). The second most frequent was using yes/no questions; however, we found two methods of asking questions: yes/no questions and WH questions. Yes/no questions were used 84 times (21%), followed by another type of question, WH questions, which were used 45 times (11%). Expressions of suffering were noted, followed by expressions of feelings. These strategies were used 32 (8%) and 28 times (7%), respectively. Another type was making a request using direct advice, followed by asking the doctor to look at the medical report/x-ray. These strategies were used 19 (5%) and 13 times (3%), respectively. Although the number of postings in the data was greater for female patients and they employed more strategies, the male patients used more expressions of feelings and asked the doctor to look at the medical report/x-ray more often. In addition, female and male patients were equal in their use of expressions of suffering.

However, the middle part, which is the main component of advice-seeking due to its frequent occurrence, has two main types: one is related to describing the problem, while the other is related to requesting advice. The first part, which describes the problem, is divided into three parts: describing the medical problem in general or describing the problem by expressing suffering or feelings. However, for the other part, which is related to requesting advice, the advice was requested in four ways: by using yes/no questions, using WH questions, using direct advice, or asking the doctor to look at the medical reports/x-ray.

#### 5.2.2. Types of compound strategies employed by patients in advice-seeking

**Figure 1:**
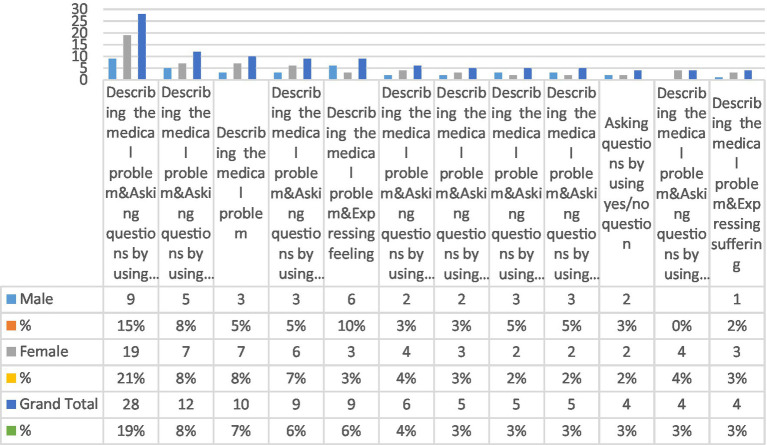
Types of compound strategies used by patients in advice-seeking.

Figure 1 has been prepared to identify the most frequent types of compound strategies used by patients seeking advice. These compound strategies were arranged from the most to the least frequent. The results revealed 48 linguistic patterns (See Appendix B for the rest of the compound strategies). However, only the most frequent types of compound strategies used four times and above in advice-seeking will be explained.

Although describing a medical problem was the most frequent strategy, it was not always used alone, as shown in Figure 1. Describing the medical problem and using yes/no questions was the most frequent compound strategy, as it was used by 19% of patients. The second most frequent type was describing the medical problem and using WH questions, followed by the third type, which involves a description of the medical problem. These strategies were used 8 and 7%, respectively. The fourth and fifth types of compound strategies were equally used (6%). For instance, the fourth type involves a description of the medical problem, asking questions using yes/no questions, and asking questions using a WH question. The fifth type involves a description of medical problems and expressions of feelings. The sixth type involved describing the medical problem, asking questions using a WH question, and expressions of suffering, which was used 4% of the time. However, the rest of the compound strategies were employed only 3% of the time. Although the compound strategies were used more by female patients than by male ones, it was observed that the male patients used the strategy of describing the medical problem by expressing feelings more than female patients (10 and 3%, respectively). In addition, the eighth and ninth types of compound strategies were used more frequently by male patients than by female ones. However, the difference was slight.

#### 5.2.3. Types of strategies employed by doctors in advice-giving

Table 5 presents the types of strategies employed by doctors. It is clear that the construction of advice-giving has three main parts: opening, middle, and closing. It is also clear that opening and closing were occasionally used, which is similar to the construction of seeking advice in Table 4. In other words, there were similarities in terms of the opening and closing parts. As can be seen, the opening includes a greeting/greeting response, such as *assalamu alaykum/wa ^c^alaykum assalam* (peace be upon you/peace be upon you), which was used six times (2%), while the use of an address term occurred only five times (2%). Although a greeting was used 19 times by patients, a greeting response or greeting was used six times by doctors. This means that greetings did not always receive a similar response in this context. Regarding the closing part, it is obvious that the use of prayers/religious expressions or wishes occurred five times (2%), as in *allah yishfi:k* (may Allah heal you) or *ʔatmanna laka ashshifaʔ al^c^ajil* (get well soon) while an expression of thanks was used only one time. This section was closed using expressions of wishes rather than thanks.

**Table 5:** Types of strategies employed by doctors.

Types of strategies in advice-giving by doctors	Male	%	Female	%	Grand total	%
Opening						
Greeting/greeting response	6	3%		0%	6	2%
Address terms	5	2%		0%	5	2%
Middle						
Giving clarification/ information	97	41%	18	35%	115	40%
Direct advice	88	37%	19	37%	107	37%
Yes/no answer	14	6%	3	6%	17	6%
Hedge advice	11	5%	4	8%	15	5%
Positive regard	9	4%	1	2%	10	3%
Giving a link for more information	1	0%	4	8%	5	2%
Asking for more clarification	4	2%		0%	4	1%
Closing						
Using prayers/religious expressions/wishes	4	2%	1	2%	5	2%
Thanks		0%	1	2%	1	0%
Grand total	239	100%	51	100%	290	100%

In the middle part, giving clarification/information was the most frequent strategy, followed by giving direct advice. These strategies were used 115 (40%) and 107 times (37%), respectively. The first two types were the most common, as they were used with high frequency compared to the others. Yes/no answers were used 17 times (6%), followed by hedge advice, which was used 15 times (5%), and positive regard, which was used 10 times (3%). Giving a link to more information was used five times (2%), followed by asking for more clarification, which was used four times (1%), making it the least frequently used strategy.

#### 5.2.4. Types of compound strategies used by doctors in advice-giving

**Figure 2:**
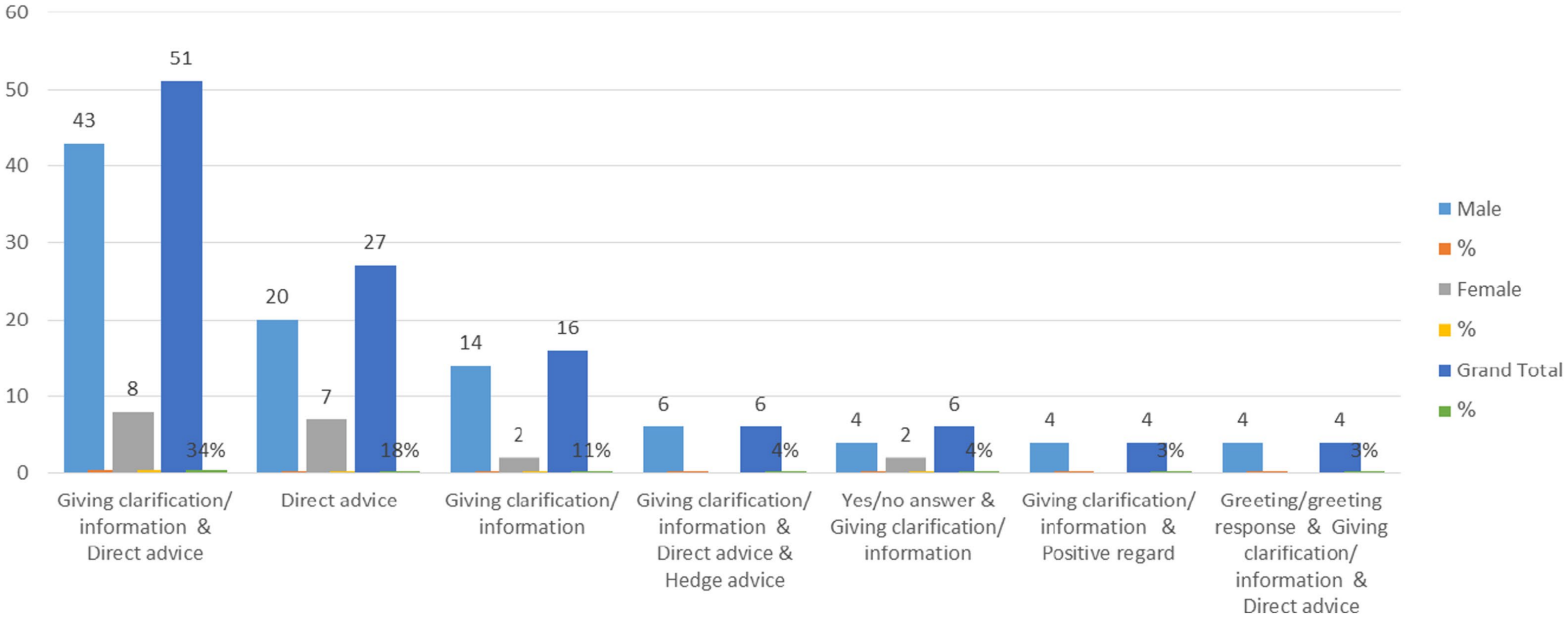
The types of compound strategies used by doctors in advice-giving.

This section clarifies the most frequently employed types of compound strategies used by doctors. These strategies were arranged from most to least frequent. Researchers identified 30 patterns of compound strategies. However, only the first seven types of compound strategies, which have a higher frequency in advice-giving, are displayed in Figure 2 (see Appendix C for the remaining types of compound strategies).

Although the strategy of giving clarification/information was the most frequent in Table 5, it was not employed alone. However, it was mostly used with direct advice. Therefore, Figure 2 shows that giving clarification/information and direct advice was the most common type of compound strategy used by doctors in advice-giving, as it was used 51 times (34%). The second most frequent pattern was giving direct advice, followed by clarification. These strategies were used 27 (18%) and 16 times (11%), respectively. It also clarifies that the fourth type, which involves clarification/information, direct advice, and hedge advice, as well as the fifth, which includes a yes/no answer and giving clarification/information, were used with the same frequency 6 (4%). In addition, the sixth, giving clarification/information and positive regard, and the seventh, a greeting/greeting response, giving clarification/information, and direct advice, were used with the same frequency, 4 (3%), making them the least frequently used compound strategies.

## 6. Discussion

The current study aimed to examine the discourse patterns of advice-seeking and advice-giving in Arabic to identify strategies and their construction through CMC in the medical context. The findings revealed that advice-seeking and advice-response messages have three parts: opening, middle, and closing. However, not all of these parts can be found in all messages. It was noticed that both patients and doctors used the opening and closing parts occasionally, and they are considered positive politeness strategies in terms of greetings and farewells or thanks. When a closing is used by doctors, it expresses empathy and positive regard. This is unlike the middle part, which was most frequently used. It can be said that the reason behind using an opening and closing depends slightly on the nature of the context, which is CMC, particularly due to the physical absence of doctors when patients are seeking advice. This is unlike FTF communication, where patients mostly use greetings as an opening (e.g., hello) or messages of thanks as a closing (e.g., thank you) because of the physical presence of the doctor at the time of interaction.

It was also found that 142 (36%) patients described their medical problem, making it the most frequent type of advice-seeking. This finding is inconsistent with Kouper’s (2010) study, which showed that requests for information or opinions are the most common type of advice solicitation. However, this is similar to Morrow’s (2006) study in the health domain on an Internet discussion forum, which showed that the description of the problem was the main part of advice-seeking. The intended meaning in describing the problem can be understood by doctors as patients asking for advice (Decapua and Dunham, 1993). Although describing the problem was the second most frequently used strategy, it was used mostly through asking questions, either the yes/no or WH type. However, more yes/no questions were used than WH questions. Questions are considered a request for advice. The use of yes/no questions can be interpreted as implying that the patient has some competence in dealing with his/her medical problem (Heritage and Sefi, 1992; Morrow, 2006). Additionally, it has been noted that patients’ descriptions of their problems sometimes included descriptions of feelings and/or suffering that came in the form of affirmative and negative statements among male and female patients (e.g., I suffer from dizziness, I do not suffer from.., I feel.., or I do not feel..). These descriptions were remarkable for their frequent use, and this is indicative of an important function that enables a patient to describe his/her problem to a doctor in an easily understandable way. The findings in Morrow’s (2006) study pointed out that the use of expressions of feelings was found in the data. However, the authors of the study did not find suffering expressions in advice-seeking. These differences could be due to the nature of the symptoms, as well as the nature of the patient-doctor interaction in the Arabic context.

Additionally, the results indicated that asking questions using yes/no questions and describing the medical problem was the most frequent compound strategy used by patients seeking advice. Moreover, it was noted that yes/no questions were used more often than WH questions in advice-seeking, which may indicate a preference in using the types of questions besides describing the problem to show competence of dealing with the patient’s problem, as mentioned above. It was also observed that some patients used more than one type of WH question, and they sometimes used yes/no questions with them. This finding is in line with Kouper’s (2010) study, which showed that one message often contains several questions and supports the findings of Pung (2017). Another thing to note is that doctors sometimes do not respond to yes/no questions with yes/no answers, as they also use clarifications. It could be that doctors want to ensure that patients understand their answers clearly due to the nature of the context, which is related to people’s health. In addition, brief answers such as yes/no answers are not adequate in the medical context, especially for patients with whom the doctors are not familiar. It has also been noted that some patients ask for advice on behalf of others (e.g., my son), which occurred occasionally, emphasizing the ease of seeking advice for anyone and at any time for any symptom. These expressions were used normally due to it being a context where young children cannot ask for themselves. Although the number of postings was not equal for the male and the female patients, the former used expressions of feelings or asked the doctor to look at the medical reports/x-rays more than the latter. In addition, the genders were equal in expressing suffering. In terms of the compound strategies, the male patients described the medical problem and expressed feelings more than the female ones. Some patients uploaded an x-ray and asked doctors to look at it to interpret the results. However, it was not clear why the patient did not ask his/her doctor, or what the reason was for seeking advice in an online context, not through face-to-face communication; in particular, the x-ray was usually requested by a doctor, and the result was interpreted to the patient in a follow-up appointment.

For advice responses, on the other hand, the results showed similarity in being less frequently used in opening and closing parts in advice responses for the same reason, unlike the middle part. In addition, giving clarification/information was the most frequent strategy, followed by direct advice. Doctors used imperatives when the medical problem was obvious to them and the patient needed to take immediate action based on the doctor’s advice. However, hedge advice was observed with possible actions that the patient might take or when the medical problem was not obvious to the doctors or there was a doubt about giving direct advice. Most hedges were used in the strategy of giving clarification, not in the advice itself, as Hinkel (1997, 2005) found. In contrast, Bates (2021) found that the use of hedges was the most common strategy used by advice-seekers, not advice-givers. Providing clarification/information and direct advice were used remarkably often compared to other types. Kouper (2010) arrived at a similar conclusion in the analysis of advice interactions in an online community, which showed that the most common form of advice used was direct advice, followed by sharing of personal experiences. However, Kouper’s (2010) study is inconsistent with the current study in terms of sharing personal experiences, which could be due to the nature of the context, that is, healthcare. The results also showed that giving clarification/information and direct advice was the most frequently used compound strategy used by doctors in advice responses, as it was used 51 times (34%). It seems that doctors know that there will be no reply from patients due to the nature of the context (Altibbi), which lacks a mechanism for providing a response from patients. Therefore, doctors try to provide sufficient clarification and direct advice to ensure that patients understand them correctly. This might be the reason for the use of this type of compound strategy at a high frequency. Additionally, it was revealed that prayers/religious expressions were used in advice. This result is supported by El-Dakhs’ (2021) study, which showed that the Saudi Health Department used more religious expressions in advice-giving. In addition, Hosni (2021) strongly agreed with this result when finding these expressions in his study in Arabic. Therefore, it can be said that these expressions are used because of Islamic principles. Moreover, asking for more clarification by doctors occurred only four times (1%). This pattern is used when a doctor wants to know more information about a patient’s condition. However, there was no reply by patients because of the nature of the website, which had two parts: advice-seeking and advice responses. No later comments were available for the patients. Morrow (2006) considered asking questions by advisory givers as indirect advice. It has also been found that advice-givers employed positive regard strategies (e.g., do not worry) for advice-seekers to help them feel optimistic about their case. This finding is supported by Morrow’s (2006) study, which showed that this strategy was used to communicate positively. In addition, it was observed that the verb “advise” (e.g., I advise you…) in the current data was used 12 times. This finding supports the findings of Hosni’s (2021) study, which claims that the verb “advise” was found to be used in the Arabic data but was never used performatively in the English part. It can be said that these differences occur because of cultural differences or the context itself.

## 7. Conclusion

The current study aimed to identify the discourse patterns and strategies of advice-seeking, advice responses, and the construction of messages in Arabic through CMC in the medical context on the Altibbi website. Two models were used to analyze the speech acts regarding advice. Morrow’s (2006) model was used for advice-seeking, while Hinkel’s (1997) taxonomy was used for advice responses with some modifications to both models according to the data. Therefore, the results showed that the types of advice-seeking and advice-response strategies had three parts: opening, middle, and closing. It was noted that both patients and doctors used the opening and closing parts occasionally, unlike the middle part, which was used more frequently. For advice-seeking, the results revealed that describing the medical problem was the most frequent strategy used by patients when they asked for advice, followed by asking yes/no questions. These two types were used in advice-seeking more frequently than other types of strategies. Additionally, it was found that using yes/no questions and describing the medical problem was the most frequent compound strategy used by patients seeking advice. However, for advice responses, the results showed that giving clarification/information was the most frequent strategy used by doctors, followed by giving direct advice. When compared to other types, the first two were used frequently in the advice responses. In addition, the results showed that giving clarification/information and direct advice was the compound strategy most frequently used by doctors in advice responses.

The implications of these findings can help doctors and clinics understand the language used by patients when seeking advice or requesting medical consultation in an online context, because doctors give advice based on the patient’s description of the symptoms and questions, not based on a clinical examination. Some patients feel comfortable seeking advice in an online context when the medical problem is related to something that is not taboo in society, such as symptoms or medical problems related to illegal relationships and the use of drugs. Websites for medical consultations need to give patients a chance to respond to the doctor’s response, as the patient’s inability to provide only one posting constrains the interaction between the patient and doctor. In some responses, it was found that doctors ask for more clarification or provide a link to patients for more information about the symptoms. In other words, it is better to give the patient a chance to interact with the doctor and enable them to better understand their medical case by responding to the doctor’s questions, which would give patients a chance to follow up with questions. The findings can benefit online medical consultations in terms of helping patients understand the language used by doctors when giving advice, which would improve the service for patients, whether the advice was sufficient or not, because the consultation is based on the oral descriptions provided by patients, not a clinical examination.

One limitation of this study is that it aimed to investigate advice-seeking by patients and advice responses by doctors. There was no focus on patients’ comments after they received doctors’ advice because the option to post follow-up questions was not available to the patients. Therefore, further research should include more sequences following the advice given by doctors. In addition, for future research, it is recommended to examine gender and age differences in advice-seeking in CMC in Arabic, because the data were limited in this study.

## Data availability statement

The original contributions presented in the study are included in the article/Supplementary material, further inquiries can be directed to the corresponding author.

## Author contributions

NA-M and MM contributed to conception and design of the study, manuscript revision, read, and approved the submitted version. NA-M organized the database, wrote the introduction, literature review, and discussion with conclusion of the manuscript. MM performed the statistical analysis, and wrote the methodology and the analysis of the manuscript. All authors contributed to the article and approved the submitted version.

## Conflict of interest

The authors declare that the research was conducted in the absence of any commercial or financial relationships that could be construed as a potential conflict of interest.

## Publisher’s note

All claims expressed in this article are solely those of the authors and do not necessarily represent those of their affiliated organizations, or those of the publisher, the editors and the reviewers. Any product that may be evaluated in this article, or claim that may be made by its manufacturer, is not guaranteed or endorsed by the publisher.

## Supplementary material

The Supplementary material for this article can be found online at: https://www.frontiersin.org/articles/10.3389/fpsyg.2023.1070310/full#supplementary-material
