# Supplementary material for: Advice-seeking and advice-giving in Arabic computer-mediated communication in the medical context
Source: Front Psychol. 2023 Sep 22;14:1070310. doi: 10.3389/fpsyg.2023.1070310 (PMC10557946; doi:10.3389/fpsyg.2023.1070310)
Supplement: Supplementary file 1 [file Data_Sheet_1.pdf]

Appendix A: Transliteration symbols for Arabic vowels and consonants

Arabic Letter	English Symbol	Arabic Example	English Equivalent
ء	ʔ	ʔamal	hope
ب	b	ba:b	door
ت	t	tibn	chaff
ث	th	tha^{c}lab	fox
ج	j	jamal	camel
ح	h̲	h̲ub	love
خ	kh	khubz	bread
د	d	dub	bear
ذ	dh	dhahab	gold
ر	r	rab	Lord
ز	z	zayt	oil
س	s	sabt	Saturday
ش	sh	shams	sun
ص	s̲	s̲ayf	summer
ض	d̲	d̲ayf	guest
ط	t̲	t̲i:n	mud
ظ	TH	THuhr	noon
ع	^{c}	^{c}abd	slave
غ	gh	gharb	west
ف	f	fam	mouth
ق	q	qalam	pencil
ك	k	kita:b	book
ل	l	layl	night
م	m	makr	guile
ن	n	nawm	sleep
هـ	h	hudhud	hoopoe
وَ	w	ward	rose
يَ	y	yawm	day
َ (فتحة)	a	kataba	he wrote
ُ (ضمة)	u	kutub	books
ِ (كسرة)	i	sin	tooth
مد طويل ا/ى	a:	ka:tib	writer
ضمة طويلة و	u:	fu:l	beans
كسرة طويلة ي	i:	fi:l	elephant
Diphthongs	aw	mawt	death
(أصوات علة مركبة)	ay	bayt	House

Source: Retrieved and adapted from http://www.ijaes.net/Author/Help.

3 Appendix B: Types of compound strategies in advice-seeking by patients

	Types of Compound Strategies in advice-seeking	Male	%	Female	%	Grand Total	%
1	Describing the medical problem&Asking questions by using yes/no question	9	15%	19	21%	28	19%
2	Describing the medical problem&Asking questions by using WHQ	5	8%	7	8%	12	8%
3	Describing the medical problem	3	5%	7	8%	10	7%
4	Describing the medical problem&Asking questions by using yes/no question&Asking questions by using WHQ	3	5%	6	7%	9	6%
5	Describing the medical problem&Expressing feeling	6	10%	3	3%	9	6%
6	Describing the medical problem&Asking questions by using WHQ&Expressing suffering	2	3%	4	4%	6	4%
7	Describing the medical problem&Asking questions by using yes/no question&Expressing feeling	2	3%	3	3%	5	3%
8	Describing the medical problem&Asking questions by using yes/no	3	5%	2	2%	5	3%

	question&Expressing suffering						
9	Describing the medical problem&Asking questions by using yes/no question&Greeting	3	5%	2	2%	5	3%
10	Asking questions by using yes/no question	2	3%	2	2%	4	3%
11	Describing the medical problem&Asking questions by using yes/no question&Asking the doctor to look at the medical reports/x&ray		0%	4	4%	4	3%
12	Describing the medical problem&Expressing suffering	1	2%	3	3%	4	3%
13	Asking questions by using WHQ&Describing the medical problem&Expressing feeling	2	3%	1	1%	3	2%
14	Describing the medical problem&Requesting direct advice	2	3%	1	1%	3	2%
15	Asking questions by using WHQ&Asking questions by using yes/no question&Describing the medical problem&Expressing suffering		0%	2	2%	2	1%
16	Asking questions by using yes/no question&Describing the medical problem&Expressing		0%	2	2%	2	1%

	feeling&Expressing suffering						
17	Asking questions by using yes/no question&Describing the medical problem&Expressing suffering &Requesting direct advice&Using prayers	1	2%	1	1%	2	1%
18	Asking questions by using yes/no question&Describing the medical problem&Requesting direct advice		0%	2	2%	2	1%
19	Asking questions by using yes/no question&Using address term&Describing the medical problem		0%	2	2%	2	1%
20	Describing the medical problem&Expressing feeling&Expressing suffering	1	2%	1	1%	2	1%
21	Greeting&Asking questions by using WHQ&Asking questions by using yes/no question&Describing the medical problem		0%	2	2%	2	1%
22	Greeting&Asking questions by using WHQ&Describing the medical problem&Expressing suffering	1	2%	1	1%	2	1%
23	Greeting&Asking questions by using yes/no question&Describing the medical		0%	2	2%	2	1%

	problem&Expressing feeling						
24	Asking questions by using WHQ&Asking questions by using yes/no question&Describing the medical problem&Requesting direct advice		0%	1	1%	1	1%
25	Asking questions by using WHQ&Describing the medical problem&Expressing suffering &Requesting direct advice&Asking the doctor to look at the medical reports/x&ray	1	2%	0	0%	1	1%
26	Asking questions by using WHQ&Describing the medical problem&Requesting direct advice		0%	1	1%	1	1%
27	Asking questions by using WHQ&Describing the medical problem&Requesting direct advice&Using prayers		0%	1	1%	1	1%
28	Asking questions by using WHQ&Describing the medical problem&Thanking	1	2%	0	0%	1	1%
29	Asking questions by using WHQ&Requesting direct advice&Asking the doctor to look at the medical reports/x&ray	1	2%	0	0%	1	1%

30	Asking questions by using yes/no question&Describing the medical problem&Expressing feeling&Expressing suffering &Asking the doctor to look at the medical reports/x&ray	1	2%	0	0%	1	1%
31	Asking questions by using yes/no question&Describing the medical problem&Requesting direct advice&Thanking		0%	1	1%	1	1%
32	Asking questions by using yes/no question&Describing the medical problem&Thanking		0%	1	1%	1	1%
33	Asking questions by using yes/no question&Requesting direct advice&Using prayers		0%	1	1%	1	1%
34	Asking questions by using yes/no question&Using address term&Describing the medical problem&Expressing suffering &Asking the doctor to look at the medical reports/x&ray	1	2%	0	0%	1	1%
35	Asking questions by using yes/no question&Using address term&Expressing feeling&Requesting direct advice&Asking the doctor to look at the medical		0%	1	1%	1	1%

	reports/x&ray&Using prayers						
36	Describing the medical problem&Expressing feeling&Asking the doctor to look at the medical reports/x&ray	1	2%	0	0%	1	1%
37	Describing the medical problem&Expressing feeling&Expressing suffering &Asking the doctor to look at the medical reports/x&ray	1	2%	0	0%	1	1%
38	Describing the medical problem&Requesting direct advice&Asking the doctor to look at the medical reports/x&ray	1	2%	0	0%	1	1%
39	Describing the medical problem&Requesting direct advice&Using prayers	1	2%	0	0%	1	1%
40	Greeting&Asking questions by using WHQ&Describing the medical problem&Expressing feeling&Expressing suffering	1	2%	0	0%	1	1%
41	Greeting&Asking questions by using WHQ&Describing the medical problem&Requesting direct advice		0%	1	1%	1	1%
42	Greeting&Asking questions by using WHQ&Describing the medical	1	2%	0	0%	1	1%

	problem&Requesting direct advice&Thanking						
43	Greeting&Asking questions by using yes/no question&Describing the medical problem&Thanking		0%	1	1%	1	1%
44	Greeting&Asking questions by using yes/no question&Thanking		0%	1	1%	1	1%
45	Greeting&Asking questions by using yes/no question&Using address term&Describing the medical problem		0%	1	1%	1	1%
46	Greeting&Describing the medical problem		0%	1	1%	1	1%
47	Greeting&Describing the medical problem&Expressing suffering	1	2%	0	0%	1	1%
48	Using address term&Describing the medical problem&Expressing suffering &Asking the doctor to look at the medical reports/x&ray	1	2%	0	0%	1	1%
	**Grand Total**	**59**	**100%**	**91**	**100%**	**150**	**100%**

Appendix C: Types of compound strategies in Advice-giving by doctors

	**Types of Compound strategies in Advice-giving by doctors**	**Male**	**%**	**Female**	**%**	**Grand Total**	**%**
1	Giving clarification/ information & Direct advice	43	35%	8	30%	51	34%
2	Direct advice	20	16%	7	26%	27	18%
3	Giving clarification/ information	14	11%	2	7%	16	11%
4	Giving clarification/ information & Direct advice & Hedge advice	6	5%		0%	6	4%
5	Yes/no answer & Giving clarification/ information	4	3%	2	7%	6	4%
6	Giving clarification/ information & Positive regard	4	3%		0%	4	3%
7	Greeting/greeting response & Giving clarification/ information & Direct advice	4	3%		0%	4	3%
8	Giving clarification/ information & Direct advice & Positive regard	3	2%		0%	3	2%
9	Yes/no answer	3	2%		0%	3	2%
10	Address term & Giving clarification/ information & Direct advice	2	2%		0%	2	1%
11	Giving clarification/ information & Religious expressions/using prayers/Wishing	2	2%		0%	2	1%
12	Giving clarification/ information & Direct advice & Giving a link for more information		0%	2	7%	2	1%
13	Giving clarification/ information & Direct advice & Asking for more clarification	2	2%		0%	2	1%
14	Giving clarification/ information & Hedge advice	1	1%	1	4%	2	1%
15	Yes/no answer & Giving clarification/ information & Direct advice	2	2%		0%	2	1%
16	Yes/no answer & Giving clarification/ information & Hedge advice	2	2%		0%	2	1%
17	Yes/no answer & Direct advice	2	2%		0%	2	1%
18	Address term & Giving clarification/ information	2	2%		0%	2	1%
19	Address term & Giving clarification/ information & Direct	1	1%		0%	1	1%

	advice & Asking for more clarification						
20	Direct advice & Hedge advice		0%	1	4%	1	1%
21	Giving clarification/ information & Giving a link for more information	1	1%		0%	1	1%
22	Giving clarification/ information & Positive regard & Religious expressions/using prayers/Wishing		0%	1	4%	1	1%
23	Giving clarification/ information & Direct advice & Religious expressions/using prayers/Wishing	1	1%		0%	1	1%
24	Giving clarification/ information & Direct advice & Hedge advice & Giving a link for more information		0%	1	4%	1	1%
25	Giving clarification/ information & Direct advice & Hedge advice & Positive regard	1	1%		0%	1	1%
26	Giving clarification/ information & Hedge advice & Giving a link for more information		0%	1	4%	1	1%
27	Greeting/greeting response & Asking for more clarification	1	1%		0%	1	1%
28	Greeting/greeting response & Giving clarification/ information & Direct advice & Positive regard	1	1%		0%	1	1%
29	Yes/no answer & Thanking		0%	1	4%	1	1%
30	Yes/no answer & Giving clarification/ information & Hedge advice & Religious expressions/using prayers/Wishing	1	1%		0%	1	1%
	Grand Total	123	100%	27	100%	150	100%
